# Diol-enhanced natural deep eutectic solvents for efficient poplar pretreatment

**DOI:** 10.3389/fchem.2026.1790752

**Published:** 2026-04-29

**Authors:** Chaehwi Yoon, Jiae Ryu, Soyeon Jeong, Aymerick Eudes, Kwang Ho Kim, Chang Geun Yoo

**Affiliations:** 1 Department of Chemical Engineering, State University of New York Environmental Science and Forestry, Syracuse, NY, United States; 2 Feedstocks Division, Joint BioEnergy Institute, Emeryville, CA, United States; 3 Environmental Genomics and Systems Biology Division, Lawrence Berkeley National Laboratory, Berkeley, CA, United States; 4 Department of Wood Science, University of British Columbia, Vancouver, BC, Canada; 5 Institute for Sustainable Materials and Manufacturing, State University of New York Environmental Science and Forestry, Syracuse, NY, United States

**Keywords:** 3,4-dihydroxybenzoic acid, diols, fractionation, natural deep eutectic solvents, viscosity

## Abstract

Natural deep eutectic solvents (NDESs) are promising biomass pretreatment media, but their industrial application is often hindered by high viscosity. To address this limitation, diol-enhanced ternary DESs (TDESs) were prepared by incorporating 1,4-butanediol (1,4-BDO) or ethylene glycol (EG) into a choline chloride (ChCl) and 3,4-dihydroxybenzoic acid (DHBA) system. The applied TDESs maintained a liquid state at room temperature and had significantly reduced viscosity compared to the binary DES (BDES). In addition, the applied diols increased lignin solubility and suppressed lignin condensation by intercepting reactive carbocation intermediates. As a result, the recovered lignins from diol-induced TDES pretreatments showed better preservation of β-O-4 linkages and reduced condensation, improving their potential for downstream valorization. The diol-assisted DES systems showed a synergistic effect from the reduced viscosity, enhanced lignin solubility, and suppression of unwanted condensation, resulting in more effective biomass pretreatment performance, including the enhanced delignification and higher enzymatic digestibility compared to BDES. The 1,4-BDO-enhanced DES was also successfully applied to DHBA-enriched transgenic poplar, highlighting its potential for the processing of engineered biomass feedstocks.

## 1 Introduction

Lignocellulosic biomass is abundant, environmentally friendly, and a promising carbon-neutral alternative to petroleum-derived resources. Structurally, it consists of cellulose, hemicellulose, and lignin, which are strongly connected through lignin-carbohydrate complexes (LCCs). To overcome biomass recalcitrance caused by intrinsic structural complexity and heterogeneity of the lignocellulosic biomass, pretreatment is applied (Dong et al., 2019). During pretreatment, LCCs are disrupted, breaking the intrinsic interactions within biomass, enabling effective fractionation and utilization of each component.

Recent efforts in biomass pretreatment solvent design have focused on achieving high conversion efficiency and minimal side reactions, as well as renewability and/or recyclability to enable a sustainable processing system (Kim and Yoo, 2021). Deep eutectic solvents (DESs) have been introduced as green and tunable solvents for many applications, including biomass pretreatment. DESs are eutectic mixtures composed of hydrogen bond acceptors (HBAs), such as quaternary ammonium salts, and hydrogen bond donors (HBDs), such as acids, amines, carboxylic acids, polyols, and aromatic compounds (Abbott et al., 2004; Florindo et al., 2018; Kim et al., 2018). Hydrogen bonding interactions between HBAs and HBDs reduce the lattice energy, leading to the melting point depression (Smith et al., 2014). By combining diverse HBAs and HBDs, a wide range of DESs can be synthesized with tunable physicochemical properties, including phase behavior, ionic conductivity, viscosity, and thermal stability (Hansen et al., 2021).

Natural deep eutectic solvents (NDESs) have been developed using components derived from natural resources (Mamilla et al., 2019). Among NDESs, DESs composed of aromatic constituents have great potential to enable circular and closed-loop biorefinery strategies through the utilization of biomass-derived aromatics, particularly from the fractionated lignin. Lignin is an abundant aromatic polymer in secondary plant cell walls, which can be extracted through biomass pretreatment, followed by further conversion into useful aromatic compounds. Previous studies have shown that DESs incorporating lignin-derived aromatics, such as *p*-coumaric acid, vanillin, *p*-hydroxybenzoic acid, and phenolic aldehydes as HBDs, exhibit effective biomass fractionation performance (Kim et al., 2018; Ryu et al., 2024; Wang et al., 2020; Wang et al., 2023; Ryu et al., 2025). 3,4-Dihydroxybenzoic acid (DHBA) is also a potential lignin-derived HBD containing carboxylic and hydroxyl functional groups (Milenković et al., 2017; Shmonova et al., 2025). These functional groups can provide active protons, thereby facilitating hydrogen bonding of DES and promoting delignification (Yu et al., 2022). Additionally, Unda et al. reported that 3,4-dihydroxybenzoate can be incorporated into the lignin backbone of poplar via plant genetic engineering (Unda et al., 2022), providing a foundation for integrating feedstock engineering with circular biorefinery strategies. However, these DESs incorporating lignin-derived aromatic compounds have relatively high viscosity, which limits mass and heat transfer during reactions, and often solidify at room temperature, thereby reducing pretreatment performance and processability (del Mar Contreras-Gámez et al., 2023). To overcome these limitations, recent studies have introduced a third component, such as water, ethylene glycol, or organic solvents, to conventional binary DESs (Sima et al., 2021). In a previous study, Wang et al. investigated choline chloride (ChCl)-DHBA-based DESs for sorghum stover pretreatment and reported that the addition of water improved pretreatment performance (Wang et al., 2023). However, water-induced ChCl-DHBA DES was solidified at room temperature, making the recovery of carbohydrate-rich solid challenging after the DES pretreatment. In addition, the presence of water increased the volatility of the DES under biomass pretreatment conditions, diluting one of the main advantages of DESs: their low operating pressure. Furthermore, while the role of diol incorporation in suppressing lignin condensation has been discussed (Cheng et al., 2022), its influence on solvent processability, including viscosity, thermal stability, and phase transition behavior, has not been comprehensively investigated. Therefore, an integrated understanding of solvent properties and fractionation performance is needed.

In this study, 1,4-butanediol (1,4-BDO) was introduced to improve the pretreatment performance of ChCl-DHBA DES for poplar by reducing solvent viscosity and preventing solidification. Ethylene glycol (EG) was also applied as a reference diol component. Furthermore, lignin stabilization was expected with the applied diols based on previous studies (Li et al., 2022; Liu et al., 2021; Wang et al., 2022). ChCl was selected as an HBA because of its non-toxicity, biodegradability, and effectiveness in biomass pretreatment (Jose et al., 2023). The physicochemical properties of the DESs, including phase transition temperatures, thermal degradation, and viscosity, were analyzed to elucidate the effect of diol introduction into ChCl-DHBA DES. Pretreatment performance was evaluated by assessing changes in chemical composition and enzymatic hydrolysis of the DES-pretreated biomass. In addition, structural properties and molecular weight distribution of fractionated lignins were analyzed to determine their potential in post-utilization approaches. The feasibility of a sustainable solvent system using 1,4-BDO-enhanced ChCl-DHBA DES was also discussed, along with its pretreatment application to DHBA-enriched transgenic poplar.

## 2 Materials and methods

### 2.1 Materials

The wild-type poplar (20–40 mesh) obtained from Oak Ridge National Laboratory was used as the primary feedstock to assess various ChCl-DHBA DES pretreatments. The chemical composition of the untreated poplar was 49.9% glucan, 21.3% xylan, 26.5% lignin, and 0.8% ash. Additionally, the *QsuB*-poplar Line 1 grown at Lawrence Berkeley National Laboratory was tested as a feedstock to evaluate the applicability of the selected pretreatment to transgenic poplar (Unda et al., 2022). Alcell lignin, obtained from Oak Ridge National Laboratory, was used for the qualitative lignin solubility test. ChCl (98%) and DHBA (98%) obtained from Sigma-Aldrich were used as HBA and HBD, respectively. 1,4-BDO and EG from Sigma-Aldrich were also used to investigate the effects of diol constituents in DHBA-DESs. Microcrystalline cellulose (99%) was obtained from Alfa Chemistry. Cellulase blend (Cellic® CTec2), sodium acetate trihydrate (99%), and sodium azide (99%) were also obtained from Sigma-Aldrich. Cellulase enzyme activity was measured according to the NREL protocol and determined to be 58.8 FPU mL^-1^ (Adney et al., 2008).

### 2.2 Synthesis of DHBA-DESs

To prepare the binary ChCl-DHBA DES, ChCl and DHBA were mixed at 120 °C for 3 h in a 3:2 molar ratio, as reported previously (Wang et al., 2023). 1,4-BDO or EG was added to the ChCl-DHBA DES, with molar ratios of 3:2:1 or 3:2:2 for ChCl, DHBA, and diols in the ternary DESs (TDESs), respectively. Table 1 summarizes the abbreviations of the prepared DESs with different compositions.

**TABLE 1:** The molar composition of the prepared DHBA-DESs.

ChCl	DHBA	Diols		Abbreviation
3	2	–	0	BDES
3	2	1,4-BDO	1	TDES-B1
3	2	1,4-BDO	2	TDES-B2
3	2	EG	1	TDES-E1
3	2	EG	2	TDES-E2

### 2.3 Characterization of DESs

The phase transitions of ChCl-DHBA DESs were identified by utilizing differential scanning calorimetry (DSC, TA Instrument Discovery DSC 250). To prepare the DSC analysis, 3–5 mg of fully dried DES samples were loaded into a T-zero hermetic pan and sealed with a T-zero hermetic lid. To eliminate the thermal history of the samples, two consecutive heating-cooling cycles were performed at a heating rate of 3 °C min^-1^. The temperature was scanned from −80 °C–120 °C. The measurements were conducted under a nitrogen atmosphere.

Thermal degradation temperatures of ChCl-DHBA DESs were analyzed by thermogravimetric analysis (TGA, TA Instrument Discovery TGA 550). Around 5–10 mg of dried ChCl-DHBA DES samples were placed into a T-zero hermetic pan and covered with a pinhole lid. The samples were heated to 500 °C at a rate of 10 °C min^-1^. The measurements were carried out under a nitrogen atmosphere.

The rheological properties of DHBA-DESs were characterized using a rheometer (TA Instrument HR20) equipped with a 25 mm parallel plate geometry and a Peltier plate. The viscosity was measured over a shear rate range of 1–100 s^-1^ at 140 °C.

### 2.4 DES pretreatments

The poplar sample was loaded into a Pyrex bottle containing the prepared binary or ternary ChCl-DHBA DESs at a 1:10 (w/w) solid-to-liquid ratio. The pretreatment conditions were based on our previous study (Wang et al., 2023). In brief, the DES pretreatments were performed in Pyrex bottles at 140 °C for 3 h with continuous stirring. Once the pretreatment was completed, an ethanol/water solution (1:1, v/v) was added to immediately quench the reaction and cool to room temperature. The pretreated poplar was filtered under vacuum to obtain a solid residue, which was then further washed with an ethanol/water solution.

### 2.5 Chemical composition, crystallinity, and morphological analyses of untreated and pretreated poplar

The chemical compositions of untreated and pretreated poplar were determined based on the NREL protocol (Sluiter et al., 2008). Specifically, biomass samples were soaked in 72 wt% sulfuric acid at 30 °C for 1 h in a water bath while stirring the samples with a regular glass rod. After the reaction, the samples were diluted to 4 wt% acid with deionized water and autoclaved at 121 °C for 1 h. The hydrolysate was filtered through glass filters, and the solid was dried in a 105 °C oven to determine Klason lignin, accounting for the remaining inorganic content in the lignin. The filtered hydrolysate was used to analyze acid-soluble lignin by measuring the absorbance at 240 nm wavelength using a UV-vis spectrometer. Furthermore, the hydrolysate was filtered through a 0.2 μm nylon syringe filter and then analyzed by high-performance liquid chromatography (HPLC, Agilent Technologies 1260 Infinity) using a Bio-Rad Aminex HPX-87H column, with a RefractoMax 520 refractive index (RI) detector. Ash content was determined by combusting ∼1 g of the sample at 575 °C for 24 h in a muffle furnace.

The crystallinity of untreated and pretreated biomass was determined by powder X-ray diffraction (XRD) using a Bruker D2 Phaser diffractometer equipped with a LynxEye 1D silicon strip detector and Cu Kα radiation (λ = 1.5418 Å). XRD patterns were obtained from a 5°–50° range of 2θ with 0.05° steps and a counting time of 0.5 s per step. The crystallinity index (CrI) was calculated using the Segal method. The intensity of the (002) diffraction peak (I_002_, 2θ = 22.5°) and the minimum intensity between the (110) and (002) peaks, corresponding to the amorphous contribution (I_AM_, 2θ = 18.7°), were used in the calculation (Park et al., 2010).

To examine the morphological properties of untreated and pretreated samples, scanning electron microscopy (SEM, JEOL JSM IT-100) analysis was conducted. Before analysis, all samples were vacuum-dried to remove residual moisture. Subsequently, samples were mounted on metal stubs and sputter-coated with gold and palladium. The SEM images were taken at an accelerating voltage of 10 kV.

### 2.6 Enzymatic hydrolysis of poplar

Enzymatic hydrolysis of untreated and pretreated poplar was conducted at a solid loading of 1 wt% in 50 mM sodium acetate buffer containing 0.02% sodium azide at pH 4.95. The loading of cellulases was adjusted to 15 FPU g^-1^ of biomass. Enzymatic hydrolysis was performed in an incubator at 50 °C and 200 rpm for 120 h. Aliquots were periodically collected and heated to 95 °C to quench the hydrolysis reaction and then filtered through a 0.22 μm nylon syringe filter for HPLC analysis.

### 2.7 Characterization of physicochemical properties of lignin

Cellulolytic enzyme lignin (CEL) was obtained to represent native lignin from untreated poplar. Poplar was ball-milled, and then enzymatic hydrolysis was carried out. Subsequently, 96% dioxane extraction, centrifugation, and freeze-drying were performed on the solid residues, as described in the previous study (Yoo et al., 2016). Lignin samples were also recovered from the liquid phase after ChCl-DHBA DES pretreatments. In brief, lignin was precipitated by the addition of water to the DES pretreatment solvent at low temperature (4 °C), followed by additional washing with water and freeze-drying.

The structural properties of the obtained lignins were analyzed by ^1^H–^13^C two-dimensional heteronuclear single quantum coherence nuclear magnetic resonance (2D HSQC NMR) using a Bruker AVANCE III 600 MHz NMR spectrometer. Dimethylsulfoxide-*d*_*6*_ (DMSO-*d*_*6*_) was used as an NMR solvent. The HSQC results were obtained with the following acquisition parameters: 12 ppm of spectral width in the F2 (^1^H) dimension with 512 data points, 160 ppm of spectral width in the F1 (^13^C) dimension with 1024 data points, 32 of the total number of scans, and 1.2 s of relaxation delay.

The weight-average molecular weight (M_w_), number-average molecular weight (M_n_), and dispersity (*Đ*) of lignins were measured by gel permeation chromatography (GPC, Waters 2489 GPC system) equipped with Waters Styragel columns (Waters Corporation, Milford, MA) and a UV detector at 254 nm. A calibration curve was generated using standard polystyrene samples (500–50000 g mol^-1^). The lignins were acetylated in a pyridine/acetic anhydride mixture before the analysis. The prepared acetylated lignin was dried with a rotary evaporator and then dissolved in tetrahydrofuran (THF). The obtained sample was filtered through a polytetrafluoroethylene (PTFE) syringe filter for the GPC analysis.

The lignin solubility test was conducted by loading 40 mg of Alcell lignin into 1 mL of solvent and vortexing. The samples were centrifuged at 14000 rpm for 20 min. The supernatant was removed, and the solid was vacuum-dried and weighed for comparison.

### 2.8 Analysis of the degree of polymerization of cellulose

α-Cellulose was isolated to determine the degree of polymerization (DP) of cellulose in untreated and DES-pretreated poplar. To obtain α-cellulose, biomass samples were treated with an aqueous 32% peracetic acid solution to isolate holocellulose. Subsequently, deionized (DI) water was used to wash the holocellulose sample by centrifuging until the pH reached neutral. A two-step treatment with 17.5% and 8.75% sodium hydroxide solutions was performed on the recovered holocellulose to isolate α-cellulose. The prepared α-cellulose was converted to cellulose tricarbanilate using anhydrous pyridine and phenyl isocyanate at 70 °C for 48 h. Methanol was used to quench the reaction. Furthermore, a methanol/water solution was used for precipitation. Derivatized cellulose tricarbanilate was dissolved in THF and filtered through a 0.45 μm PTFE syringe filter, and weight-average degree of polymerization (DP_w_), number-average degree of polymerization (DP_n_), and *Đ* were further analyzed using GPC (Waters 2489 GPC system) with a UV detector at 254 nm. The DP_w_ and DP_n_ were calculated as M_w_/M_0_ and M_n_/M_0_, respectively, where M_w_ and M_n_ were obtained from GPC and M_0_ is the monomeric weight of derivatized cellulose (M_0_ = 519 g mol^-1^). A calibration curve was generated using standard polystyrene (100000–3580000 g mol^-1^).

## 3 Results and discussion

### 3.1 Effects of the diols on characteristics of ChCl-DHBA DESs

The characteristics of DESs are crucial for discussing their processability and biomass pretreatment performance. The properties of DESs can be tailored by selecting the solvent composition. In this study, the molar ratio of 3:2 of ChCl to DHBA was adopted from previous studies (Wang et al., 2023) based on biomass pretreatment efficiency. The molar ratios of the selected ChCl and DHBA with diols were adjusted to 3:2:1 and 3:2:2 based on our preliminary study.

Measuring the melting point depression relative to the individual components is essential for verifying the eutectic point of DESs. Compared with the melting temperature (T_m_) of individual ChCl (301 °C) and DHBA (221 °C), binary DES (BDES) exhibited a decreased T_m_ to 87 °C, confirming the successful formation of DES. After introducing diols, in TDESs, phase transition temperatures, including T_m_, glass transition temperature (T_g_), and crystallization temperature (T_cc_), were not clearly observed, except for TDES-B1 (T_g_ at −63.8 °C) (Table 2; Supplementary Figure S1). The TDESs existed in a liquid state at room temperature; thereby, the solidification of BDES at room temperature was solved with these TDESs.

**TABLE 2:** Phase transition temperatures (T_g_, T_cc_, T_m_) and onset degradation temperature (T_onset_) of ChCl-DHBA DESs.

DESs	T_g_ (°C)	T_cc_ (°C)	T_m_ (°C)	T_onset_ (°C)
BDES	−49.5	22.4	87.4	288.3
TDES-B1	−63.8	Not detected	Not detected	276.0
TDES-B2	Not detected	Not detected	Not detected	250.1
TDES-E1	Not detected	Not detected	Not detected	260.5
TDES-E2	Not detected	Not detected	Not detected	216.3

The thermal degradation behavior of DESs indicates their stability during biomass pretreatment. Table 2; Supplementary Figure S2 show the thermal degradation profiles of the binary and ternary ChCl-DHBA DESs. The onset degradation temperature (T_onset_) of BDES was highest (288 °C) and exhibited a decrease in TDESs, resulting in the following order: TDES-B1 (276 °C) > TDES-E1 (261 °C) > TDES-B2 (250 °C) > TDES-E2 (216 °C). As the molar ratio of diols increased, T_onset_ gradually decreased. In addition, TDESs containing 1,4-BDO showed higher thermal stability than those containing EG Both BDES and TDESs with ChCl and DHBA exhibited T_onset_ above 210 °C, indicating that they were thermally stable at the pretreatment temperature used in this study (140 °C). These results demonstrate that ChCl-DHBA DES pretreatments were conducted at a thermally appropriate temperature.

**FIGURE 1:**
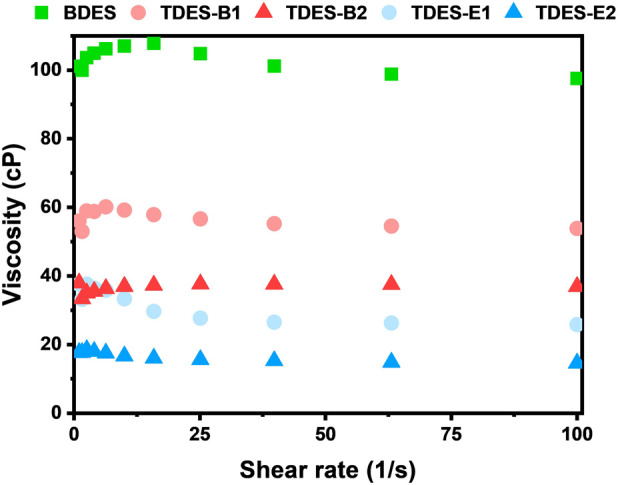
Viscosity of ChCl-DHBA DESs in the shear rate range of 1–100 s^-1^.

Viscosity has been reported as a crucial property of DESs, determining solvent fluidity and mass transfer during biomass pretreatment (Wang et al., 2023). A lower solvent viscosity results in greater molecular mobility, facilitating solvent penetration into inter-fibrillated biomass structures and encouraging intermolecular collisions (Ee et al., 2023). Figure 1 presents the viscosity behavior of ChCl-DHBA DESs measured at 140 °C, corresponding to the biomass pretreatment temperature in this study. BDES exhibited a relatively high viscosity of 98 cP at 100 s^-1^, while the viscosities of TDESs were significantly decreased: TDES-B1 (54 cP) > TDES-B2 (37 cP) > TDES-E1 (26 cP) > TDES-E2 (15 cP). The results indicate that the introduction of both diols significantly reduced the viscosity of BDES, improving solvent fluidity and mass transfer characteristics.

### 3.2 Effects of the diols on ChCl-DHBA DES pretreatment performance

**FIGURE 2:**
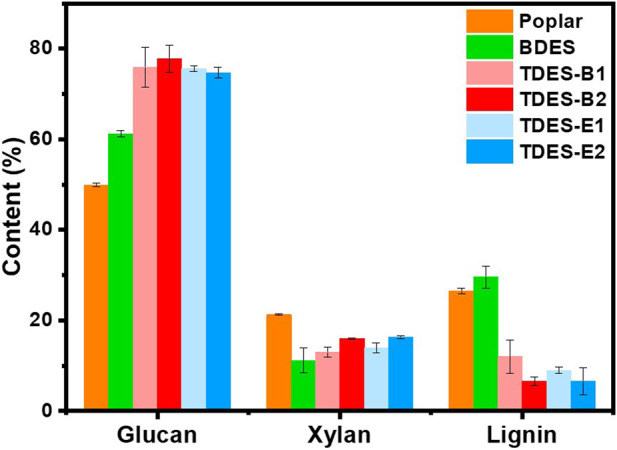
Chemical compositions of untreated and ChCl-DHBA DES pretreated poplar samples.

**FIGURE 3:**
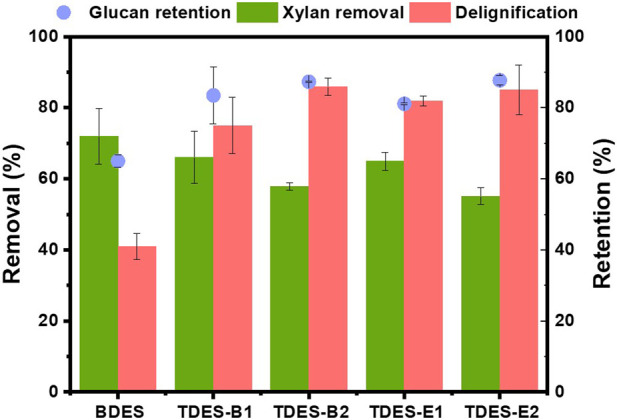
Pretreatment performance of ChCl-DHBA DESs (glucan retention, xylan removal, delignification).

For efficient glucan utilization, it is beneficial to obtain feedstock containing high glucan content after pretreatment. As shown in Figure 2, the glucan content increased from 49.9% in untreated poplar to 61.2% in BDES and further to 74.7%–77.9% in TDESs. This indicates that feedstock with a high glucan content can be obtained by effectively removing other components during the pretreatments. On the other hand, xylan content was substantially reduced by the pretreatments. Compared to untreated poplar (21.3%), the xylan content decreased to 11.2% with BDES, 13.0% and 13.9% with TDES-B1 and TDES-E1, and 16.0% and 16.3% with TDES-B2 and TDES-E2, respectively. The addition of diols slightly reduced the xylan removal performance of DES (Figure 3). This can be explained by the reduced acidity of DES as the diol molar ratio increased. The lignin content slightly increased to 29.6% after BDES pretreatment compared to untreated poplar (26.5%). This is because BDES removed more carbohydrate fractions (mainly hemicellulose) than lignin, resulting in a relative increase in lignin content. However, as the diols were introduced, the lignin content decreased significantly, resulting in the following order: TDES-B1 (12.1%) > TDES-E1 (9.0%) > TDES-E2 (6.7%) ≈ TDES-B2 (6.6%). This is attributed to the enhanced carbohydrate preservation and more efficient lignin removal as the molar ratio of diol in TDES increased. These overall results demonstrate that introducing diols into BDES can enable the production of glucan-rich feedstock with low lignin content, suitable for efficient downstream conversion.

To evaluate the fractionation performance of ChCl-DHBA DES pretreatments, glucan retention, xylan removal, and delignification were compared based on changes in chemical composition (Figure 3). Although BDES showed relatively high xylan removal (72.0%) and delignification (40.9%), glucan retention was only 65.0%. In contrast, TDESs exhibited better glucan retention, with 83.4% in TDES-B1, 81.1% in TDES-E1, 87.3% in TDES-B2, and 87.7% in TDES-E2. This enhanced glucan retention suggests that TDESs may mitigate the excessive acidity associated with BDES-mediated glucan decomposition. The TDESs also showed a decreased xylan removal to 66.1% with TDES-B1, 64.9% with TDES-E1, 57.9% with TDES-B2, and 55.1% with TDES-E2, respectively. This is consistent with the pattern of glucan retention, in which BDES showed the highest glucan loss, suggesting that the diols in the applied DESs mitigated carbohydrate removal during the pretreatments. Notably, delignification demonstrated a different pattern. The delignification of BDES pretreatment (40.9%) was significantly increased when TDESs were applied, reaching 75.0% with TDES-B1, 81.9% with TDES-E1, 86.0% with TDES-B2, and 85.3% with TDES-E2, respectively. Furthermore, delignification increased as the molar ratio of the applied diols increased. This enhancement may be attributed to decreased viscosity and enhanced lignin solubility of the DES system, which facilitates mass transfer during pretreatment (Hou et al., 2017). Along with reduced DES viscosity, enhanced lignin solubility with the applied diols also contributed to increased delignification (Du et al., 2025; Yu et al., 2019). To confirm this, Alcell lignin was subjected to water, 1,4-BDO, and EG for a solubility test (Supplementary Table S1). Consequently, lignin exhibited higher solubility in 1,4-BDO and EG than in water, indicating that the diols have the potential to enhance lignin solubility in TDESs, thereby synergistically improving delignification with reduced viscosity. Overall, TDESs showed better glucan retention and selective delignification, whereas BDES was effective for xylan removal. Thus, pretreatment using TDESs enabled selective delignification with enhanced glucan retention.

**FIGURE 4:**
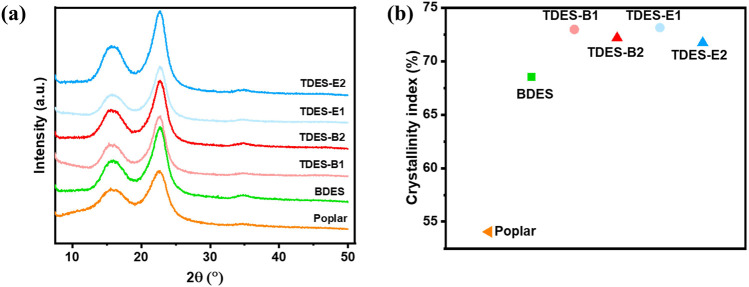
**(a)** The XRD spectra and **(b)** CrI of untreated and ChCl-DHBA DES pretreated poplar.

The CrI of untreated and pretreated poplar was determined by XRD analysis. In the previous studies, the diffraction peaks at 15.6° and 22.5° correspond to the (110) and (002) planes of cellulose I, respectively (Ma et al., 2025). These peaks were clearly observed in untreated poplar and remained unchanged after DHBA-DES pretreatment, suggesting that the recovered biomass retained the cellulose I structure (Figure 4a). As shown in Figure 4b, the untreated poplar had a CrI of 54.1%, the lowest among the samples. The CrI of the poplar sample increased to 68.5% after BDES pretreatment, and the TDES pretreatments resulted in higher CrIs (71.7%–73.1%). CrI can be influenced by biomass composition (Gümüskaya et al., 2003; Kim et al., 2010). In untreated poplar, it exhibited the lowest CrI due to its higher hemicellulose and lignin content, both of which are amorphous. The CrI of poplar increased by ChCl-DHBA DES pretreatments, mainly due to the effective removal of lignin and hemicellulose fractions (Figures 3, 4).

**FIGURE 5:**
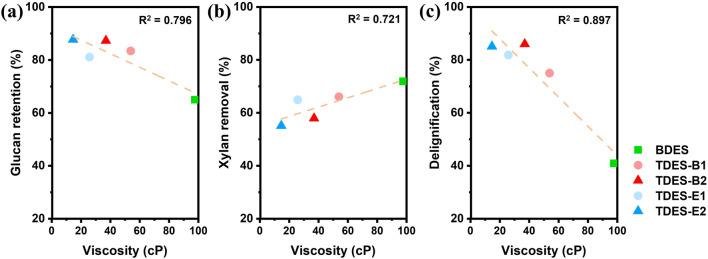
Correlations between the viscosities of ChCl-DHBA DESs and their pretreatment performance: **(a)** glucan retention, **(b)** xylan removal, and **(c)** delignification.

Overall, the introduction of diols as the third constituent significantly changed the properties of BDES, most notably by reducing viscosity, which was correlated with pretreatment performance. Figure 5 presents the correlations between the viscosity of DHBA-DESs and pretreatment performance parameters (glucan retention, xylan removal, and delignification). As viscosity decreased from BDES to TDESs, glucan retention and delignification increased, whereas xylan removal decreased. These relationships suggest that lower viscosity may have contributed to improved mass transfer and solvent fluidity, promoting penetration into the biomass matrix. However, the pretreatment performance of DES cannot be explained by a single factor like viscosity. In particular, the performance of the DESs with different diols could not be directly comparable due to other factors, such as reactivity and solubility of lignin.

### 3.3 Effects of the ChCl-DHBA DES pretreatments on the enzymatic hydrolysis of poplar

**FIGURE 6:**
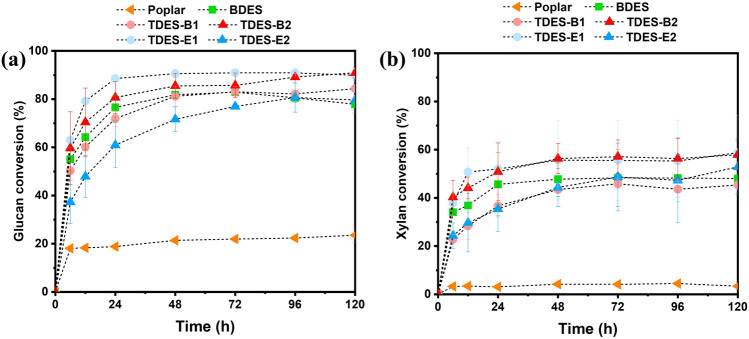
Glucan and xylan conversions of untreated and ChCl -DHBA DES-pretreated poplar:**(a)** glucan conversion and **(b)** xylan conversion.

To evaluate the pretreatment performance, the conversion efficiency of the glucan in the pretreated biomass is important. Figure 6 illustrates the enzymatic hydrolysis performance of untreated and pretreated poplar in terms of glucan and xylan conversion. Untreated poplar exhibited low glucan and xylan conversions of 23.6% and 3.4%, respectively, after 120 h of incubation. These conversions were significantly enhanced by ChCl-DHBA DES pretreatments, ranging from 77.9% to 90.9% for glucan and from 48.0% to 58.8% for xylan. The improved hydrolysis performance can be attributed to biomass disintegration, hemicellulose removal, and lignin properties during the pretreatment. The glucan conversion of BDES-pretreated poplar was improved to 77.9% by removing recalcitrance factors, such as xylan and lignin. Within the initial 24 h of hydrolysis, BDES exhibited a relatively rapid hydrolysis rate, indicating that severe structural disruption under BDES may initially improve enzyme accessibility, thereby promoting carbohydrate conversions in the early stage.

**FIGURE 7:**
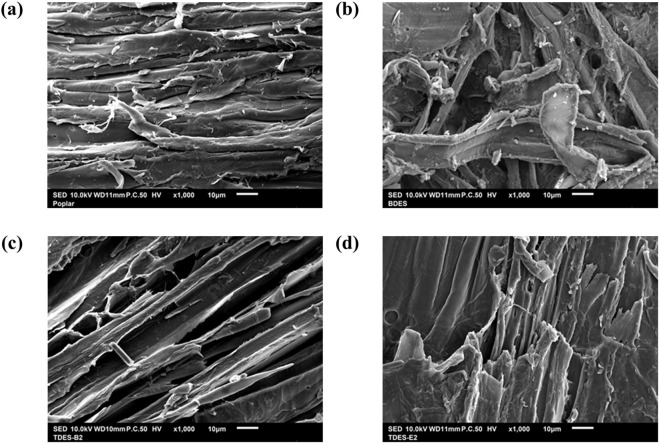
SEM images of **(a)** untreated poplar and DHBA-DES pretreated poplar from **(b)** BDES and **(c)** TDES-B2 and **(d)** TDES-E2.

TDES-B1 and TDES-B2 showed further increases in glucan conversion to 84.3% and 90.9%, respectively, as the molar ratio of 1,4-BDO increased. In particular, TDES-B2 pretreatment resulted in very low lignin content (6.6%) but the highest glucan content (77.9%), indicating that TDES-B2 is a promising pretreatment solvent based on fermentable glucose production efficiency. In TDES-E1 and TDES-E2, although the final glucan conversions were high (89.9% and 79.6%, respectively), higher EG loading exhibited a negative effect on enzymatic hydrolysis. In the previous study, similar results were reported with ChCl-oxalic acid-EG, where a high EG content in DES reduced glucan and xylan conversions in the pretreated biomass (Li et al., 2022). Although delignification with TDES-E2 was high (85.3%), its glucan digestibility was lower than that of other TDESs. These results suggest that other factors beyond lignin content affect the enzymatic hydrolysis of biomass. To understand the structural factors affecting digestibility, the morphological properties of biomass after DHBA-DES pretreatments were examined using SEM (Figure 7). As shown in Figure 7a, the untreated poplar exhibited encapsulated morphology, suggesting that cellulose accessibility was limited. Compared with untreated poplar, more fibers were exposed after DHBA-DES pretreatments, which can explain the increased enzymatic digestibility. In BDES, small particle-sized debris was observed on the fiber bundles (Figure 7b). This suggests the possible redeposition of lignin or other components onto the fiber surface during BDES pretreatment. In contrast, TDES-B2 exhibited well-exposed fibers, indicating the increased cellulose accessibility, which is associated with high glucan conversion (90.9%) (Figure 7c). In TDES-E2, some fibers were well-exposed, but others were not fully exposed (Figure 7d). At high magnification (3000 × ), small particle-sized debris was observed in BDES-pretreated biomass (Supplementary Figure S3a) and also existed in TDES-E2-pretreated biomass (Supplementary Figure S3c). However, TDES-B2-pretreated biomass exhibited a cleaner fiber surface (Supplementary Figure S3b). These observations suggest that, despite high delignification in TDES-E2, structural factors can limit enzymatic accessibility of the pretreated biomass. Therefore, this indicates that not only delignification but also the surface structural characteristics and redeposition in pretreated biomass influence enzymatic hydrolysis efficiency. Additionally, structural changes in cellulose were investigated to explain the above-mentioned results. Microcrystalline cellulose was pretreated using these DESs under the same pretreatment conditions, and their surface morphologies were examined (Supplementary Figure S4). Interestingly, TDES-E2-pretreated cellulose exhibited denser structures (Supplementary Figure S4d). This suggests that DES can influence the physical properties of cellulose, potentially influencing digestibility.

Overall, ChCl-DHBA DESs effectively alleviate biomass recalcitrance by improving carbohydrate accessibility and enzymatic hydrolysis performance. While BDES facilitates initial hydrolysis through aggressive carbohydrate disruption, TDESs provide more moderate but structurally favorable modification for carbohydrate conversion.

### 3.4 Effects of diols in ChCl-DHBA DESs on degree of polymerization of cellulose

**FIGURE 8:**
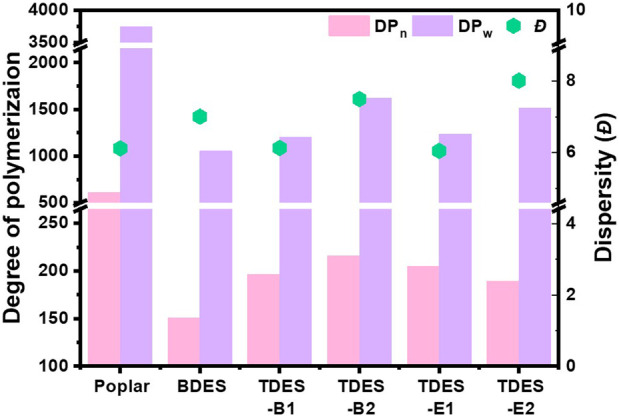
The cellulose DP and *Đ* of untreated and ChCl-DHBA DES pretreated biomass.

The cellulose DP is one of the parameters to monitor the effects of pretreatment on biomass structure and conversion, as it provides information on the availability of reducing ends in cellulose (Meng et al., 2017; Yoo et al., 2017). The cleavage of glycosidic bonds reduces the cellulose chain length and exposes more reducing ends, thereby promoting subsequent biomass conversion (Hallac et al., 2010; Meng et al., 2020; Pu et al., 2013). Figure 8 illustrates the cellulose DP and *Đ* for untreated and pretreated poplar. Untreated poplar exhibited DP_w_ and DP_n_ values of 3742 and 612, respectively, and a *Đ* of 6.1. The cellulose DP was reduced after the DHBA-DES pretreatment, suggesting cleavage of cellulose molecular chains and a decrease in molecular weight. BDES pretreatment significantly reduced DP_w_ and DP_n_ of cellulose by 71.8% and 75.4%, respectively, compared to those of the intrinsic cellulose in untreated poplar. This is consistent with the previous results showing relatively low glucan retention and a rapid hydrolysis rate during the early stage of enzymatic hydrolysis (Figure 6), suggesting more facile glycosidic bond cleavage under BDES conditions. TDES-B2 decreased the DP_w_ and DP_n_ of cellulose by 56.7% and 64.7%, respectively, compared to those in untreated poplar, demonstrating the most gradual reduction. This is consistent with the high glucan retention under TDES-B2 conditions (Figure 3). Notably, despite the preservation of relatively long cellulose chain lengths, effective enzymatic hydrolysis was achieved, suggesting that additional structural factors beyond DP reduction contributed to enhanced enzyme accessibility (Meng et al., 2020). Meanwhile, the *Đ* of cellulose showed no significant differences across the pretreatment solvents.

### 3.5 Effects of diols in ChCl-DHBA DESs on the physicochemical properties of lignin

**FIGURE 9:**
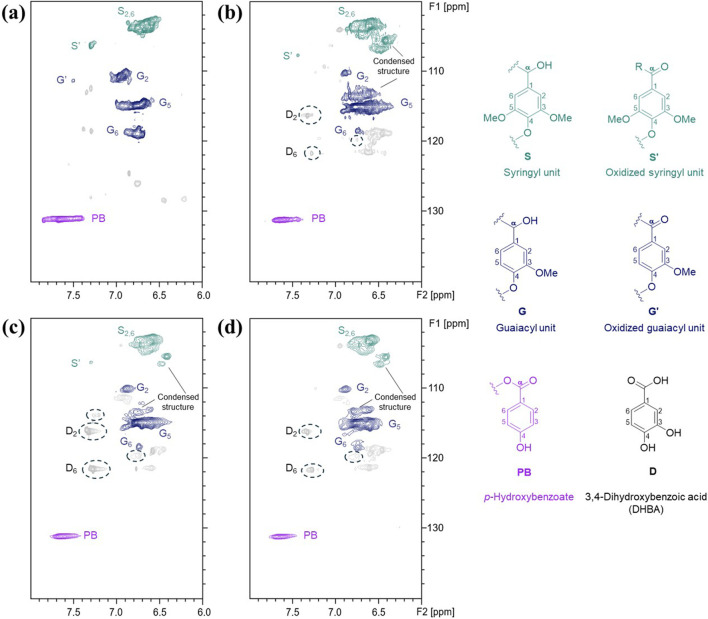
Aromatic regions of 2D HSQC NMR spectra of recovered lignin from **(a)** poplar (CEL), **(b)** BDES, **(c)** TDES-B2, and **(d)** TDES-E2. Circles indicate corresponding signals to DHBA.

**FIGURE 10:**
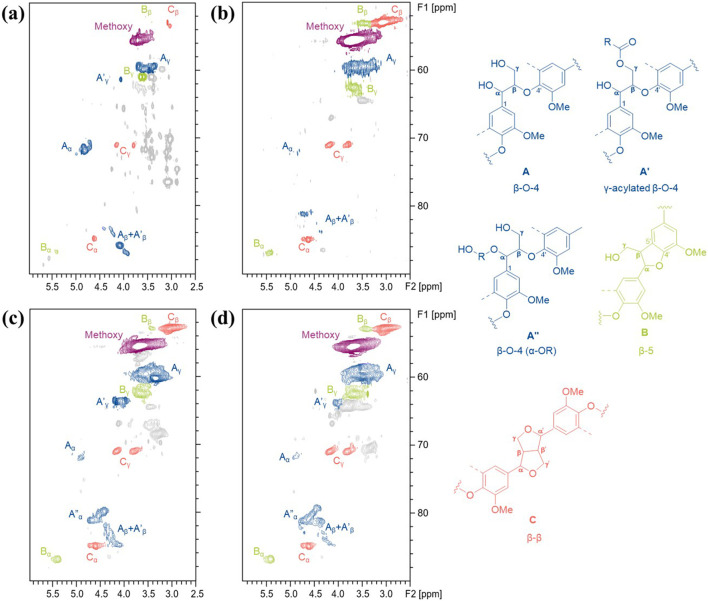
Aliphatic regions of 2D HSQC NMR spectra of recovered lignin from **(a)** poplar (CEL), **(b)** BDES, **(c)** TDES-B2, and **(d)** TDES-E2.

**FIGURE 11:**
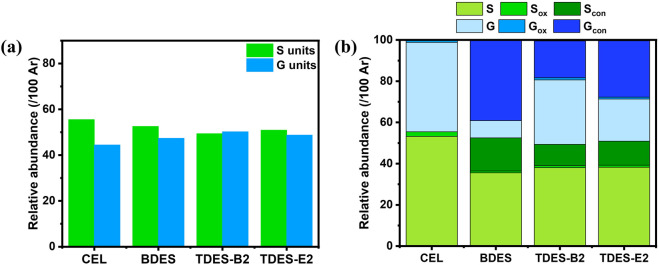
**(a)** Total S and G unit abundances in CEL and ChCl-DHBA DES recovered lignins **(b)** detailed distribution of S and G units, including non-condensed, oxidized, and condensed subunits.

**FIGURE 12:**
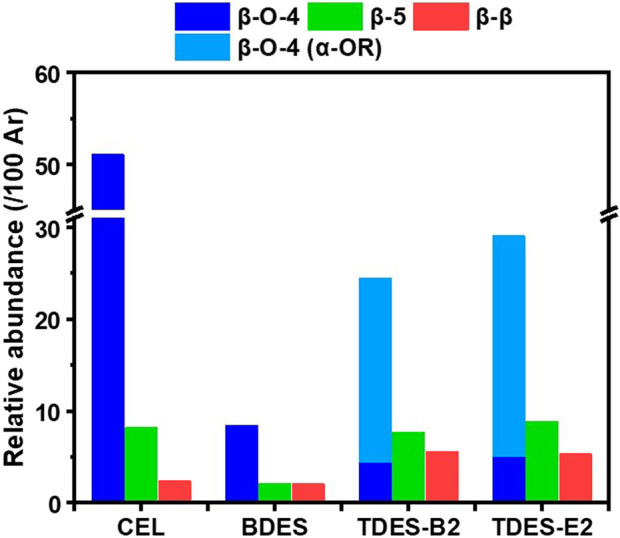
Detailed distribution of interunit linkages of CEL and ChCl-DHBA DES lignins.

Characteristics of lignin provide important information on how pretreatment transforms biomass structure, as well as clues for evaluating it as a potential co-product. Herein, aromatic unit composition, abundance of interunit linkages, and molecular weight distribution of the ChCl-DHBA DES lignins were analyzed. Based on the delignification performance, TDES-B2 lignin and TDES-E2 lignin were selected for further comparison with BDES lignin and CEL from untreated poplar. 2D HSQC NMR was conducted to demonstrate the structural characteristics and changes of the recovered lignins. NMR spectra of CEL and ChCl-DHBA DES lignins are shown in Figures 9, 10, and the cross-signals were annotated based on previous literature (Cheng et al., 2022; Wang et al., 2022). The total abundance, which was calculated as 100Ar, was based on the total integration areas of syringyl (S) and guaiacyl (G) units. In the aromatic region of lignin in Figure 9, signals of S, G units, and *p*-hydroxybenzoate structures were observed as major aromatic units. The S and G unit contents of CEL were 55.5/100Ar and 44.4/100Ar, respectively. After DES pretreatments, the total S unit content decreased, whereas the total G unit content increased slightly (Figure 11a). In terms of the S/G ratio, it slightly decreased from 1.25 (CEL) to 1.11 (BDES lignin), 0.98 (TDES-B2 lignin), and 1.04 (TDES-E2 lignin), respectively, after the DES pretreatments. Additionally, signals attributed to condensed aromatic substructures were observed in BDES lignin. This result suggests the formation of C-C linkages by condensation of some aromatic fragments during pretreatment (Shen et al., 2019). Figure 11b illustrates the detailed compositions of total S and G units, including their condensed fractions. In BDES lignin, condensed G (G_con_) and condensed S (S_con_) units were calculated as 39.0/100Ar (82.3% of total G units) and 16.0/100Ar (30.6% of total S units). However, the introduction of diols reduced the abundance of condensed aromatic subunits. In particular, in TDES-B2 lignin, the abundances of G_con_ and S_con_ decreased significantly to 17.8/100Ar (35.5% of total G units) and 10.3/100Ar (20.9% of total S units), respectively. Similarly, in TDES-E2 lignin, G_con_ and S_con_ decreased to 27.4/100Ar (56.2% of total G units) and 11.6/100Ar (22.9% of total S units). In acidic DES, lignin solubilization, depolymerization, and condensation are the major lignin reactions in previous studies (Li et al., 2021). During pretreatment, cleavage of ether linkages, especially the β-O-4 bond, plays a critical role in delignification (Cheng et al., 2022; Li et al., 2021). The acidic cleavage of aryl ether bonds is initiated by protonation of the hydroxyl group at the α-carbon of lignin. Subsequently, the α-carbon can be dehydrated, and the β-carbon can be deprotonated, generating carbocation intermediates. These intermediates can be hydroxylated at the β-carbon of lignin, resulting in β-O-4 bond cleavage (Li et al., 2021). Lignin condensation often occurs through carbocation formation via β-O-4 cleavage during pretreatments (Cheng et al., 2022). These carbocation intermediates lead to C-C condensation. However, in TDES systems, the diols in DESs play a role in forming α-etherified lignin and suppressing lignin condensation by attacking the carbocation intermediates (Liu et al., 2021). This is consistent with reduced condensed structures and increased α-etherified β-O-4 structures in TDES lignins (Figure 12). These results indicate that TDES effectively inhibited lignin condensations, suggesting that TDESs are advantageous for obtaining less-condensed lignin structures.

In the aliphatic region of the lignin HSQC spectra (Figure 10), signals corresponding to the major interunit linkages, including β-O-4, β-5, and β-β, were clearly observed. For comparison, the β-O-4 content in CEL was calculated as 51.1/100Ar. After pretreatment, the native β-O-4 contents significantly decreased to 8.4/100Ar in BDES lignin, 4.4/100Ar in TDES-B2 lignin, and 5.0/100Ar in TDES-E2 lignin. During pretreatment, carbocation intermediates can be generated by cleavage of the leaving group at the α-position of the β-O-4 structure, leading to condensation reactions (Cheng et al., 2022). However, TDES lignins showed new signals that were attributed to the grafting of 1,4-BDO or EG at the α-position of the β-O-4 linkage, as also reported in previous studies (Cheng et al., 2022; Liu et al., 2021). The diols, 1,4-BDO and EG in DESs act as nucleophiles that attack and intercept the carbocation intermediates, thereby generating α-etherified lignin and preventing condensation (Lan et al., 2018; Liu et al., 2021). In this study, the contents of preserved β-O-4 linkages (β-O-4 (α-OR)) were 20.1/100Ar in TDES-B2 lignin and 24.0/100Ar in TDES-E2 lignin (Figure 12), which were much higher than the content in BDES lignin. These results indicate that TDESs stabilized the lignin fraction by preserving a higher fraction of the β-O-4 motif than BDES. As a result, the lignin structure was shielded from further cleavage, resulting in well-preserved lignin with higher aryl ether bond content, thereby promoting the subsequent lignin valorization (Cheng et al., 2024; Wang et al., 2022). This observation is consistent with the decreased abundance of condensed subunits in the aromatic region of lignin recovered from TDES compared to BDES. Overall, these results demonstrate that TDESs maintain lignin structure more efficiently than BDES by preserving aryl ether linkages.

The molecular weight of lignin is a crucial parameter in lignin valorization and provides insight into structural changes during pretreatment (Li et al., 2017). Table 3 illustrates the M_n_, M_w_, and *Đ* of recovered lignin and CEL. Compared to CEL (M_n_: 3984 g mol^-1^, M_w_: 16733 g mol^-1^), BDES lignin exhibited a pronounced reduction in both M_n_ and M_w_, with 1101 and 1947 g mol^-1^, respectively. This result suggests extensive depolymerization of lignin, which can be attributed to the cleavage of ether linkages during the pretreatment. In contrast, in TDES lignin, M_n_ and M_w_ values were less reduced compared to BDES lignin, indicating the retention of relatively larger lignin fragments, supported by the NMR results. *Đ* of the DES lignins significantly decreased after the DES pretreatments, implying that the molecular weight distribution became more uniform.

**TABLE 3:** M_n_, M_w_, and *Đ* of lignin isolated from poplar after pretreatment using ChCl-DHBA DESs.

Lignins	M_n_ (g mol^-1^)	M_w_ (g mol^-1^)	*Đ*
CEL	3984	16733	4.2
BDES lignin	1101	1947	1.8
TDES-B2 lignin	1616	3538	2.2
TDES-E2 lignin	1510	3221	2.1

### 3.6 Effects of TDES-B2 on the pretreatment of transgenic poplar

As an extension of the previously discussed sustainable biorefinery approach, TDES-B2 was selected from the investigated TDESs and applied as a pretreatment solvent to transgenic poplar. To verify that TDES-B2 can be applied to engineered biomass, the *QsuB*-poplar Line 1, genetically engineered to accumulate DHBA, was used as a transgenic feedstock. The chemical composition of untreated and pretreated transgenic poplar is shown in Supplementary Table S2. The untreated transgenic poplar contained 41.7% glucan, 23.6% xylan, 17.6% lignin, and 2.2% ash. The pretreatment performance was shown to be 90.3% glucan retention, 61.0% xylan removal, and 72.5% delignification, indicating that TDES-B2 can be successfully applied to engineered biomass (Supplementary Table S3).

To examine the potential utilization of DHBA in the DES lignins, the presence of DHBA in the recovered lignin was observed using 2D HSQC NMR. The DHBA model compound was analyzed and used as a reference for peak identification (Supplementary Figure S5). Based on this reference, DHBA-related signals were observed in the lignin recovered from DHBA-enriched transgenic poplar after TDES-B2 pretreatment (Supplementary Figure S6). These signals can originate from residual DHBA in transgenic poplar, but they can also result from residual DES during the pretreatment process, because the signals were also detected in the aromatic regions of recovered lignin after BDES, TDES-B2, and TDES-E2 pretreatments (Figure 9). The recovered lignins after BDES, TDES-B2, and TDES-E2 pretreatments exhibited relative DHBA abundances of 1.4/100Ar, 11.4/100Ar, and 3.9/100Ar, respectively. Notably, lignin recovered from transgenic poplar after TDES-B2 pretreatment exhibited a higher relative DHBA abundance of 38.7/100Ar. This indicates that a substantial amount of DHBA recovered in the lignin fraction originates from the lignin of the transgenic poplar. Although DHBA recovery from lignin was not conducted in this study, a previous study reported that hydrothermal depolymerization can recover a high yield of aromatic compounds from engineered biomass, providing a reference for potential strategies for DHBA recovery and reusability (Kim et al., 2019). Overall, TDES-B2 was demonstrated to be applicable to engineered biomass, supporting the feasibility of integrating NDES with genetically engineered feedstocks.

## 4 Conclusion

To overcome processability challenges in renewable ChCl/DHBA DES, two diols, 1,4-BDO and EG were successfully integrated to form the ternary DESs. Both, EG and 1,4-BDO ensured that DESs remained liquid at room temperature and significantly reduced their viscosities, facilitating the biomass pretreatment process. These diols also served as chemical stabilizers, suppressing unwanted lignin condensation and promoting the preservation of β-O-4 linkages by intercepting lignin carbocation intermediates. Moreover, the enhanced lignin solubility in DESs with diols minimized the redeposition of fractionated biomass components. These synergistic effects from the applied diols improved biomass pretreatment performance. Among the formulated TDESs, TDES-B2 exhibited the most effective pretreatment performance, achieving 86.0% of delignification and 90.9% of glucan conversion. The recovered lignin exhibited a high β-O-4 linkage abundance (24.5/100Ar), which was much higher than that of BDES. Finally, the successful fractionation of DHBA-enriched transgenic poplar highlights the feasibility of the diol-enhanced DES system for a sustainable biorefinery utilizing engineered feedstocks.

## Data Availability

The original contributions presented in the study are included in the article/Supplementary Material, further inquiries can be directed to the corresponding author.
