# Supplementary material for: Diol-enhanced natural deep eutectic solvents for efficient poplar pretreatment
Source: Front Chem. 2026 Apr 29;14:1790752. doi: 10.3389/fchem.2026.1790752 (PMC13168032; doi:10.3389/fchem.2026.1790752)
Supplement: Supplementary file 1 [file DataSheet1.docx]

Supplementary Material

Diol-Enhanced Natural Deep Eutectic Solvents for Efficient Poplar Pretreatment

Chaehwi Yoon^1^, Jiae Ryu^1^, Soyeon Jeong^1^, Aymerick Eudes^2,3^, Kwang Ho Kim^4^, Chang Geun Yoo^1,5*^

^1^Department of Chemical Engineering, State University of New York Environmental Science and Forestry, Syracuse, NY, USA

^2^Feedstocks Division, Joint BioEnergy Institute, Emeryville, CA, USA

^3^Environmental Genomics and Systems Biology Division, Lawrence Berkeley National Laboratory, Berkeley, CA, USA

^4^Department of Wood Science, University of British Columbia, Vancouver, BC, Canada

^5^Institute for Sustainable Materials and Manufacturing, State University of New York Environmental Science and Forestry, Syracuse, NY, USA

**Supplementary Information**

| 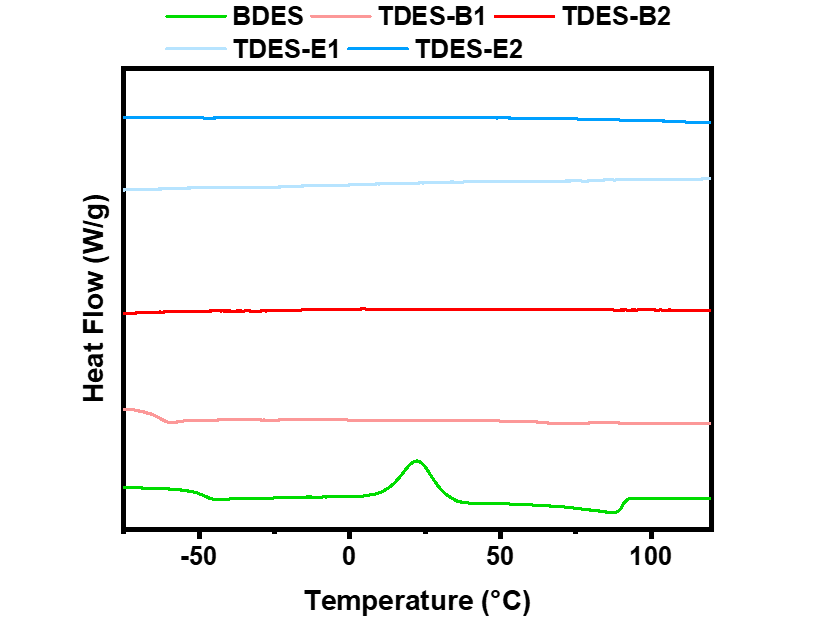 |
| --- |
| **Supplementary Figure S1.** Phase transition behavior of ChCl-DHBA DESs |

| 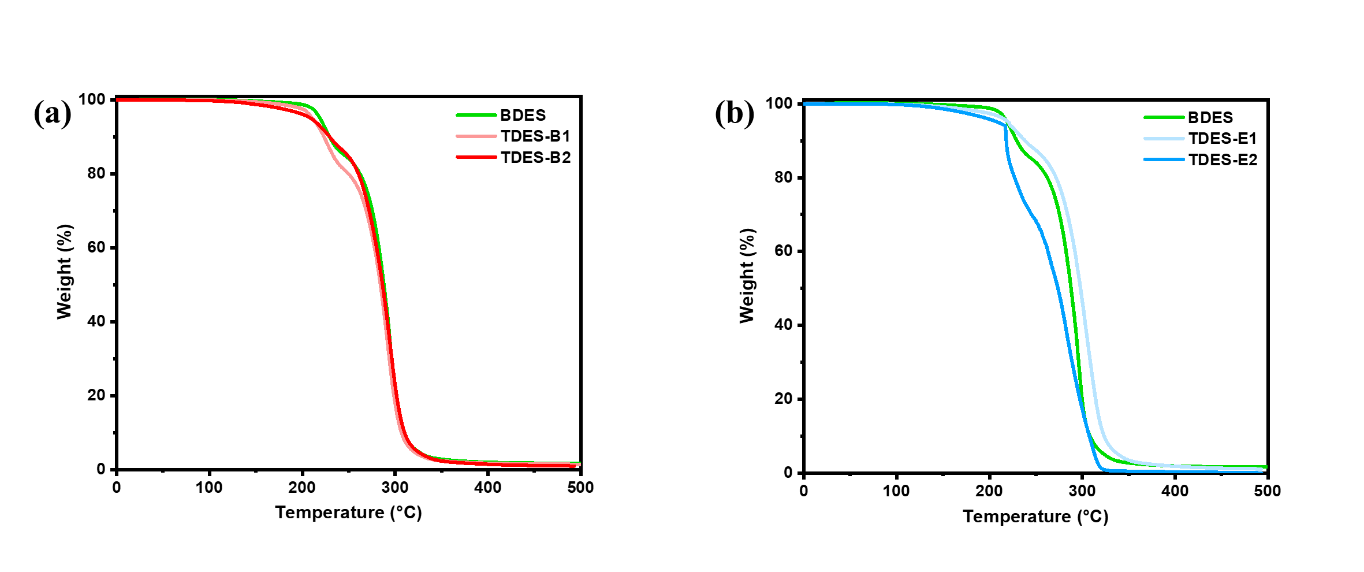 |
| --- |
| **Supplementary Figure S2.** Thermal degradation profiles of DHBA-DESs: (a) TDES-B1 and TDES-B2 and (b) TDES-E1 and TDES-E2, compared with BDES |

| 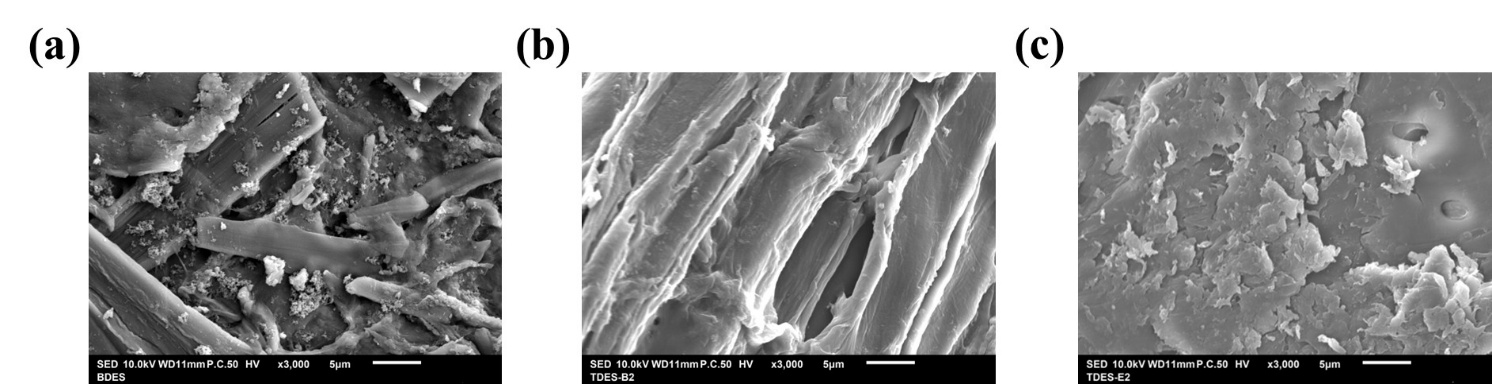 |
| --- |
| **Supplementary Figure S3.** SEM images of DHBA-DES pretreated poplar from (a) BDES and (b) TDES-B2 and (c) TDES-E2 |

| 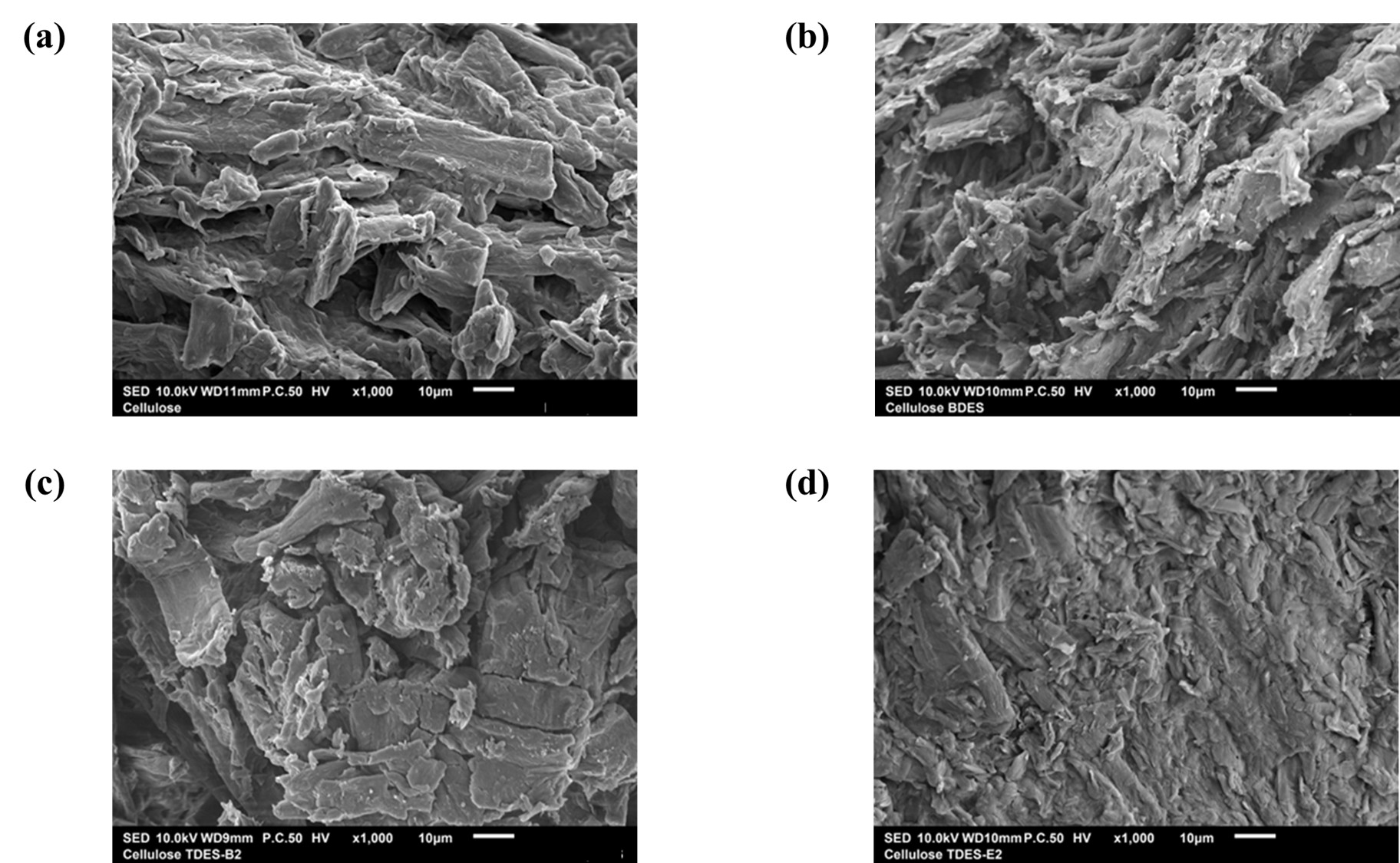 |
| --- |
| **Supplementary Figure S4.** SEM images of (a) untreated cellulose and DHBA-DES pretreated cellulose from (b) BDES and (c) TDES-B2 and (d) TDES-E2 |

| 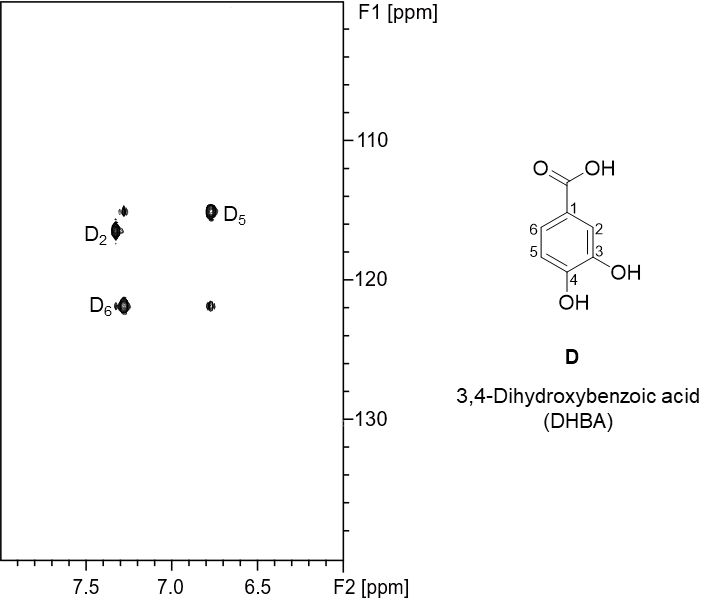 |
| --- |
| **Supplementary Figure S5.** Aromatic region of 2D HSQC NMR spectra of DHBA   \|  \| \| --- \| \| 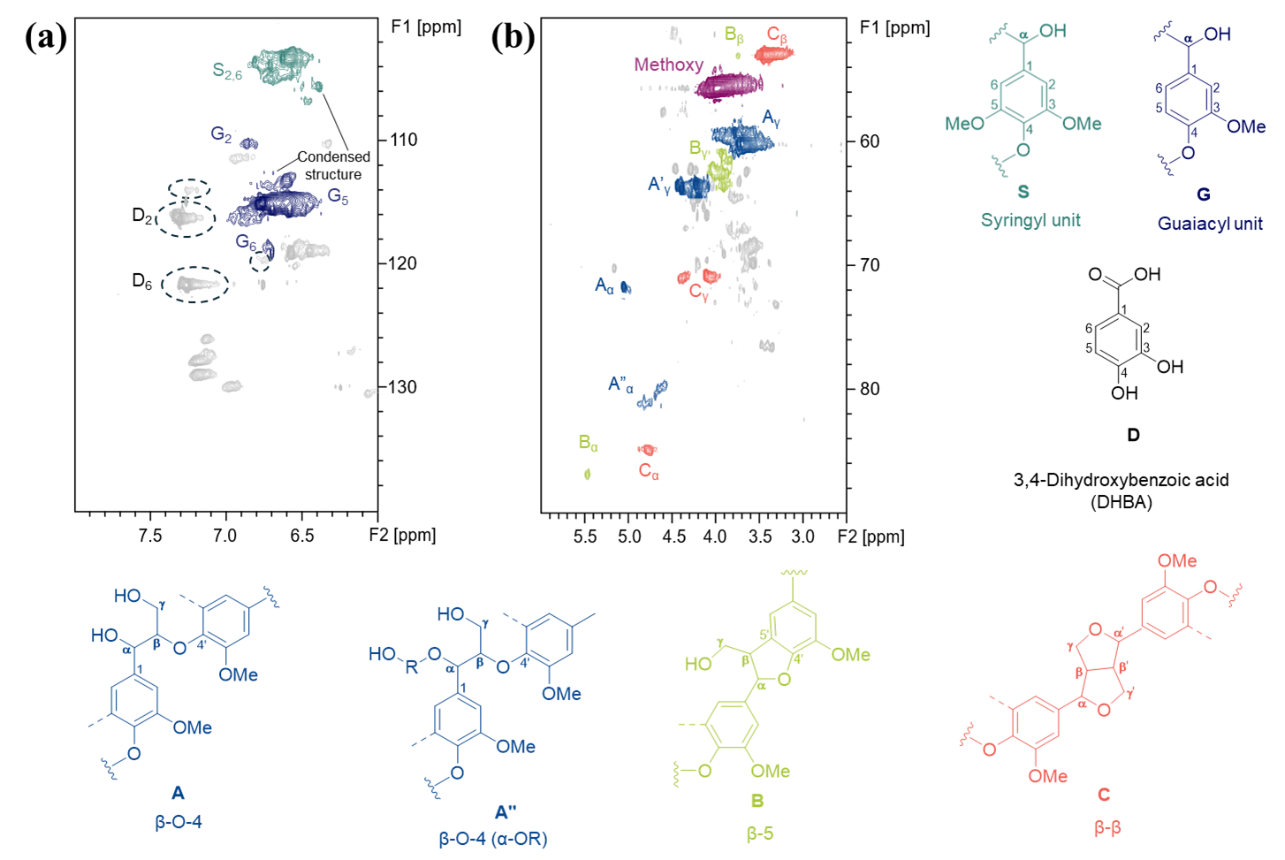 \| \| **Supplementary Figure S6.** (a) Aromatic region and (b) aliphatic region of 2D HSQC NMR spectra of recovered lignin from transgenic poplar after TDES-B2 pretreatment. Circles indicate corresponding signals to DHBA. \| |

**Supplementary Table S1.** Apparent solubility of 40 mg of Alcell lignin in different solvents

|  | **Water** | **1,4-BDO** | **EG** |
| --- | --- | --- | --- |
| **Solubility (mg mL^-1^)** | 2.5 | 36.3 | 36.6 |

**Supplementary Table S2.** Chemical compositions of transgenic poplar before and after TDES-B2 pretreatment

| **Biomass** | **Pretreatment** | **Chemical composition (%)** | | | |
| --- | --- | --- | --- | --- | --- |
|  |  | **Glucan** | **Xylan** | **Lignin** | **Ash** |
| **Transgenic Poplar** | **Untreated** | 41.7 ($\pm$0.6) | 23.6 ($\pm$0.2) | 17.6 ($\pm$1.8) | 2.2 ($\pm$0.2) |
|  | **TDES-B2** | 65.6 ($\pm$1.4) | 16.0 ($\pm$0.9) | 8.4 ($\pm$0.6) | 0.3 ($\pm$0.1) |

**Supplementary Table S3.** Pretreatment performance (glucan retention, xylan removal, delignification) of TDES-B2 applied to transgenic poplar

| **Biomass** | **Glucan retention (%)** | **Xylan removal (%)** | **Delignification (%)** |
| --- | --- | --- | --- |
| **Transgenic Poplar** | 90.3 ($\pm$2.2) | 61.0 ($\pm$2.9) | 72.5 ($\pm$1.7) |
